# Serotonin transporter density in isolated rapid eye movement sleep behavioral disorder

**DOI:** 10.3389/frsle.2023.1298854

**Published:** 2024-01-29

**Authors:** Mark Garwood, Punithavathy Vijayakumar, Nicolaas I. Bohnen, Robert A. Koeppe, Vikas Kotagal

**Affiliations:** ^1^Department of Neurology, University of Michigan, Ann Arbor, MI, United States; ^2^Division of Nuclear Medicine, Department of Radiology, University of Michigan, Ann Arbor, MI, United States; ^3^Ann Arbor Veterans Affairs Healthcare System, VAAAHS Geriatric Research Education and Clinical Center, Ann Arbor, MI, United States

**Keywords:** rapid eye movement sleep behavior disorder, serotonin, DASB, PET imaging, sleep

## Abstract

**Background/objective:**

The serotoninergic nervous system is known to play a role in the maintenance of rapid eye movement (REM) sleep. Serotoninergic projections are known to be vulnerable in synucleinopathies. To date, positron emission tomography (PET) studies using serotonin-specific tracers have not been reported in isolated REM sleep behavior disorder (iRBD).

**Methods:**

We conducted a cross-sectional imaging study using serotonin transporter (SERT) ^11^C-3-amino-4-(2-dimethylaminomethyl-phenylsulfaryl)-benzonitrile (DASB) PET to identify differences in serotonin system integrity between 11 participants with iRBD and 16 older healthy controls.

**Results:**

Participants with iRBD showed lower DASB distribution volume ratios (DVRs) in the total neocortical mantle [1.13 (SD: 0.07) vs. 1.19 (SD: 0.06); *t* = 2.33, *p* = 0.028)], putamen [2.07 (SD: 0.19) vs. 2.25 (SD: 0.18); *t* = 2.55, *p* = 0.017], and insula [1.26 (SD: 0.11) vs. 1.39 (SD: 0.09); *t* = 3.58, *p* = 0.001]. Paradoxical increases relative to controls were seen in cerebellar hemispheres [0.98 (SD: 0.04) vs. 0.95 (SD: 0.02); *t* = 2.93, *p* = 0.007)]. No intergroup differences were seen in caudate, substantia nigra, or other brainstem regions with the exception of the dorsal mesencephalic raphe [3.08 (SD: 0.53) vs. 3.47 (SD: 0.48); *t* = 2.00, *p* = 0.056] that showed a non-significant trend toward lower values in iRBD.

**Conclusions:**

Insular, neocortical, and striatal serotoninergic terminal loss may be common in prodromal synucleinopathies before the onset of parkinsonism or dementia. Given our small sample size, these results should be interpreted as hypothesis-generating/exploratory in nature.

## Introduction

Isolated Rapid eye movement (REM) sleep behavior disorder (iRBD) is an increasingly studied neurological disorder of aging that confers a high risk of conversion to a manifest neurodegenerative alpha-synucleinopathy such as Parkinson's disease (PD), Dementia with Lewy Bodies (DLB), or Multiple System Atrophy (MSA) (Zhang et al., 2022). With that said, not all individuals with iRBD will go on to progress to these conditions (Postuma et al., 2019). Developing biomarkers in iRBD that can either (1) identify patients with early manifest synucleinopathies or (2) predict phenoconversion risk to PD/DLB/MSA are of high interest to patients and clinical researchers studying sleep medicine, geriatrics, and neurodegenerative diseases.

The brain serotoninergic projection system arises from an interconnected group of raphe nuclei distributed throughout the brainstem which project rostrally to the cortex and other subcortical structures, caudally to the spinal cord, and provide autoregulatory feedback to other serotoninergic nuclei within the brainstem and cerebellum (Albert et al., 2011; Kulkarni et al., 2022). Serotoninergic neurons are known to play a role in regulating muscle tone in REM sleep. Serotoninergic drugs including selective serotonin reuptake inhibitors (SSRIs) and serotonin norepinephrine reuptake inhibitors (SNRIs) are also known risk factors for the development of REM sleep without atonia (RSWA) (Lee et al., 2016), a key component of the broader iRBD syndrome (McCarter et al., 2017).

*In vivo* nuclear medicine imaging studies of iRBD to date have used dopamine transporter ligand binding, measured within the predominantly serotoninergic raphe nuclei complex, to understand serotoninergic changes in iRBD (Arnaldi et al., 2015). Studies using a serotonin-specific tracer would offer the chance to assess serotoninergic integrity in downstream targets of the brainstem projection system. ^11^C-3-amino-4-(2-dimethylaminomethyl-phenylsulfaryl)-benzonitrile (DASB) binds to the serotonin transporter (SERT), typically present at synaptic junctions and in this way, can characterize the regional density of serotoninergic terminals (Meyer, 2007). We used DASB positron emission tomography (PET) to study serotoninergic differences between participants with iRBD and older controls.

## Methods

We conducted a cross-sectional PET imaging study of participants with iRBD. iRBD participants were identified through a retrospective chart review of potentially eligible patients with an iRBD clinical diagnosis documented in the electronic medical record system of our medical center. Each participant's chart and polysomnography (PSG) findings were then screened by a board-certified Sleep Neurologist (M.G.) to confirm the diagnosis of iRBD was consistent with the International Classification of Sleep Disorders, 3rd edition (ICSD-3) (American Academy of Sleep Medicine, 2014). In all cases, PSGs were performed for clinical indications. The average time period between PSG and the time of study visit varied across the cohort (mean days between PSG and initial study visit: 1,183.5 days, SD: 1,251.8). Eligible potential participants were then contacted by the study team to gauge their interest in participating in this observational imaging study. All participants had a face-to-face visit with the study team including a detailed neurological examination by a Movement Disorders Neurologist (V.K.) at the time of enrollment that confirmed they did not meet criteria for the diagnosis of PD, DLB, or MSA. Exclusion criteria included the following: participants with a contraindication to magnetic resonance imaging (MRI) or PET, participants with evidence of a large artery stroke or mass lesion on MRI, and participants on neuroleptic or serotoninergic drugs including SSRIs, SNRIs, tricyclic antidepressants (TCAs), bupropion, St. John's Wort (also known as Hypericum perforatum—an herbal compound with serotonin reuptake inhibitor properties), and buspirone in the 2 months preceding study enrollment. We collected and analyzed DASB PET data from a group of older healthy controls who were previously imaged at our medical center through different research protocols. This project was approved by the University of Michigan School of Medicine Institutional Review Board (UM IRBMED). All iRBD participants signed informed consent documents prior to participating. The UM IRBMED granted our study team a waiver of the United States Health Insurance Portability and Accountability Act (HIPAA) authorization to access limited retrospective DASB PET data on healthy control participants who had already undergone DASB imaging at the UM PET center in previous research studies. These healthy control participants (*n* = 16) were recruited as normal control participants and imaged with DASB PET in two different previous cohort studies studying normal aging at our center. Their ages ranged from 55 to 74 years. We sought to include control DASB imaging data in this project to offer a reference group relative to iRBD DASB findings although an important limitation is that we have limited clinical information on this control cohort.

All participants underwent brain serotonin transporter (SERT) ^11^C-3-amino-4-(2-dimethylaminomethyl-phenylsulfaryl)-benzonitrile (DASB) PET imaging. Our DASB imaging approach is described in our group's previous studies (Kotagal et al., 2012, 2018). Briefly, participants were injected with an intravenous dose of ^11^C-DASB followed by a continuous infusion delivered over 80 min (70% bolus, 30% continuous infusion). PET images were corrected for motion and were normalized into a common space using Neurostat software (https://neurostat.neuro.utah.edu). Regions of interest were defined using Brodmann areas within a Talairach atlas linked to Neurostat as well as a group of defined certain subcortical structures (Bohnen et al., 2006). Normalized *K*_1_ images were used to define regions of interest as described previously (Minoshima et al., 1994; Frey et al., 1996). We used the reference region Logan plot graphical analysis method (Logan et al., 1996) to estimate distribution volume ratios (DVRs) for regions of interest with time activity curves serving as the input function and inferior posterior cerebellar gray matter serving as the reference region for DVR calculation (Ginovart et al., 2001; Meyer, 2007). DVRs for all regions of interest were calculated as the bilateral mean of left and right hemispheres/brain regions. Total neocortical mantle DVR was calculated as the mean value across Brodmann areas (BAs), excluding BAs 13–16 (Insular cortex—defined separately) and BAs 33 and 41 (excluded due to technical or other factors). PET data on the healthy controls, analyzed differently using a separate reference region, have been reported previously as well (Chou et al., 2022). We used descriptive statistics to summarize cohort demographic and clinical factors in Table 1. We compared DASB DVRs between regions of interest in iRBD vs. healthy control participants using two sample *t*-tests. Within iRBD participants, we used Pearson's correlation coefficient to test the correlation between Montreal Cognitive Assessment (MoCA) score and DVR values in the listed regions of interest.

**Table 1:** Cohort demographic and imaging factors.

	**iRBD (*n* = 11)**	**Healthy controls (*n* = 16)**	***t*-test/chi-square; *p*-value**
Age	72.5 years (1.54)	66.0 years (1.30)	*t* = −3.24, *p* = 0.003
Sex	10 M/1 W	10 W/6 M	χ^2^ = 7.70, *p* = 0.006
Montreal cognitive assessment	26.6 (2.7)	Not available	–
Movement disorders society unified Parkinson's disease rating scale motor exam score	8.5 (4.4)	–	–
**Serotonin transporter positron emission tomography (DASB PET) distribution volume ratio (DVR) by region of interest**			
Total neocortical mantle	1.13 (0.07)	1.19 (0.06)	*t* = 2.33, *p* = 0.028
Thalamus	2.22 (0.13)	2.17 (0.17)	*t* = −0.72, *p* = 0.478
Caudate	2.17 (0.21)	2.27 (0.20)	*t* = 1.30, *p* = 0.205
Putamen	2.07 (0.19)	2.25 (0.18)	*t* = 2.55, *p* = 0.017
Insular cortex	1.26 (0.11)	1.39 (0.09)	*t* = 3.58, *p* = 0.001
Hippocampus	1.25 (0.10)	1.28 (0.12)	*t* = 0.52, *p* = 0.606
Amygdala	1.74 (0.10)	1.75 (0.17)	*t* = 0.27, *p* = 0.790
Substantia nigra	2.54 (0.37)	2.61 (0.36)	*t* = 0.50, *p* = 0.619
Dorsal raphe nucleus	3.08 (0.53)	3.47 (0.48)	*t* = 2.00, *p* = 0.056
Pons	1.60 (0.15)	1.57 (0.15)	*t* = −0.49, *p* = 0.630
Medulla	1.40 (0.22)	1.43 (0.20)	*t* = 0.42, *p* = 0.676
Cerebellar hemispheres	0.98 (0.04)	0.95 (0.02)	*t* = −2.93, *p* = 0.007

## Results

**Figure 1:**
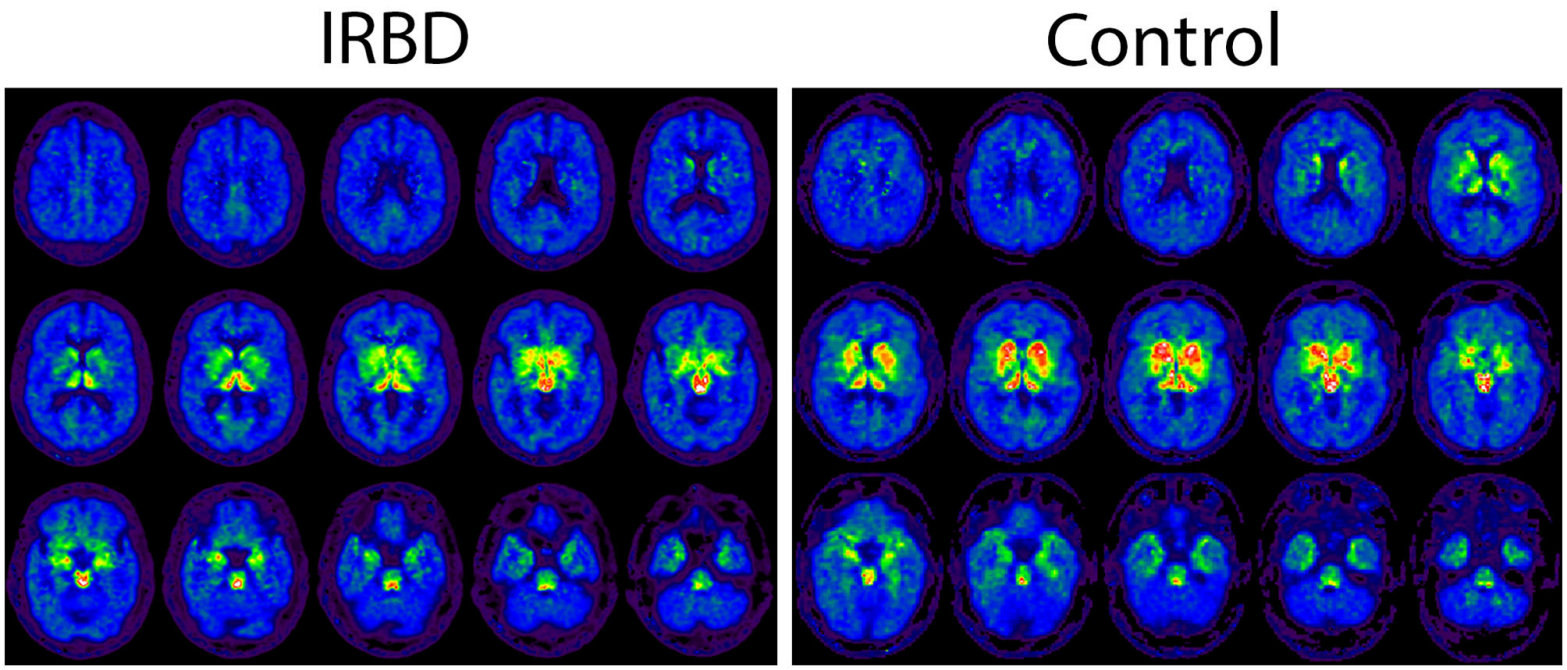
Axial DASB PET binding images depicted in a sample iRBD and control participant.

Demographic and DASB imaging factors for participants are depicted in Table 1. iRBD participants were on average older than healthy controls and more likely to be male. Decreased DASB DVR in iRBD compared to older controls, potentially reflecting loss of serotonin terminals, was seen in certain brain regions of interest including the total neocortical mantle, putamen, and insular cortex (Figure 1). A non-significant trend toward numerically lower DASB DVR in iRBD participants compared to controls was also seen in the dorsal raphe nucleus. Paradoxical relative increases in DASB DVR in iRBD participants relative to controls were seen in the bilateral cerebellar hemispheres. Within iRBD subjects, no statistically significant correlations were seen between MoCA score and DASB DVR in any of the listed regions of interest.

## Discussion

These data describe relative serotonin denervation patterns in iRBD compared to older controls most likely reflecting neurodegenerative changes. Relative reductions in iRBD serotoninergic transporter density were seen in the total neocortical mantle, the insular cortex, and the putamen. Denervation in each of these regions could conceivably be a manifestation of the earliest neurodegenerative changes suggestive of synuncleinopathies. Our cohort is relatively small, and it is worth noting that older controls in our study did differ from iRBD participants in mean age and sex. Nevertheless, these findings show a pattern of regional DASB binding differences which, if validated in larger cohorts, could serve as promising biomarkers for identifying early synucleinopathies. We also showed a modest increase in DASB binding in the cerebellar hemispheres of iRBD participants relative to controls, perhaps reflecting either brainstem-cerebellar autoregulatory functions or perhaps even favorable compensatory changes in the setting of impending neurodegeneration. It should be noted that we have limited information on our control cohort in the present study. Their underlying clinical characteristics may have influenced some intergroup DASB findings. Future studies will be needed to confirm our findings in larger prospective datasets.

Previous serotonin PET tracer studies across PD cohorts have shown region declines in 5HT projection system integrity. A DASB- PET study by Albin et al. (2008) reported a reduction in SERT binding that was most marked supratentorially in the cingulate and insular cortex and showed brainstem reductions in the pons and medulla. We previously evaluated differences in DASB PET binding in PD participants with and without symptoms of comorbid RBD and showed no significant differences between groups in raphe or striatal DASB DVR (Kotagal et al., 2012). Of note, although the software methods used to define volumes of interest differed from the present study, the mean regional DASB DVR values in both PD groups was reported in the striatum (2.27–2.30) and mean raphe nucleus (2.80–2.84). These values are somewhat higher than mean DVR values (see Table 1) in our present iRBD cohort in the striatum (caudate: 2.17 & putamen: 2.07) and lower than the DVR values seen in the dorsal raphe (3.08). These differences could potentially reflect an iRBD-specific brainstem serotoninergic imaging dysfunction that may be transient in prodromal (i.e., iRBD contexts) but that might progress rostrally as Lewy body Braak staging advances to involve the striatum over time.

Arnaldi et al. (2015) presented intergroup comparisons in a cohort of participants with iRBD and controls using ^123^I-FP-CIT single photon emission computed tomography (SPECT) imaging to assess SERT density in the brainstem and thalamus, showing no differences in binding ratios between groups in these regions. ^123^I-FP-CIT SPECT measures a combination of serotoninergic, noradrenergic, and dopaminergic terminals, rendering intergroup comparisons of small regions of interest influenced by non-serotoninergic pathology. This is especially true in the striatum where dopaminergic terminal typically predominate in healthy older adults. In light of this, our DASB findings of striatal SERT loss in iRBD present new and specific evidence for striatal serotoninergic pathology in prodromal synucleinopathies. It should also be noted that each group defined their regions of interest differently and that cohort effects might also be driving differences between our results and theirs.

Putaminal DASB DVRs were lower in iRBD participants compared to controls in our cohort. This finding could reflect impairment to the broad network of rostrally projecting terminals arising from the dorsal raphe (Miquel-Rio et al., 2023). Raphe DASB changes could also be a non-causal risk factor that just happens to be seen in individuals undergoing early striatal neurodegeneration in prodromal synucleinopathies. It is worth noting that a previous PD study has shown that DASB binding changes in the striatum manifest with a different pattern than striatal dopamine transporter imaging (Roussakis et al., 2016), suggesting that non-specific striatal changes in our iRBD cohort are not simply the earliest manifestation of nigrostriatal dopaminergic denervation. Interestingly, the Braak model of Lewy body neuropathology suggests early involvement of the medullary raphe complex but a relative sparing of the dorsal raphe from PD-related pathology (Braak et al., 2003). It is possible that region-specific dorsal raphe DASB binding reductions seen in our iRBD cohort may represent something other than monotonic neurodegeneration. It is also possible that these raphe or striatal changes are a unique signal denoting differential risk for PD vs. DLB that out cross-sectional study is not in a position to assess. Studies using multiple 5HT tracers may also be able to helpfully identify what brainstem and striatal changes are compensatory vs. degenerative.

iRBD participants in our study also showed lower neocortical and insular DASB DVRs compared to controls. Postmortem findings in parkinsonian dementias are known to involve the insular cortex where synuclein pathology has been hypothesized to be a correlate of visuospatial cognitive impairment (Yamamoto et al., 2007; Fathy et al., 2019). It has also been suggested that the insular cortex in Lewy body disorders may be a particularly vulnerable site for any number of non-synuclein neuropathologies, particularly phosphorylated tau (Fathy et al., 2022).

A 5HT1A-R PET tracer studies of the cerebral cortex in parkinsonian dementias showed correlations between high tracer regional binding and depression severity (Sharp et al., 2008) that may reflect post synaptic compensatory receptor changes in the setting of projection system deafferentation. Our cohort also showed increases in cerebellar DASB DVR in iRBD participants. It is possible that these findings represent compensatory changes. Bedard et al. (2018) have shown similar PET tracer elevations in iRBD subjects—in their case using the vesicular acetylcholine transporter ligand 18F- fluoroethoxybenzovesamical (FEOBV)—where elevations in FEOBV binding correlated with the severity of phasic and tonic electromyography (EMG) findings. Another possibility is that regional SERT expression may be autoregulated in certain highly connected brain regions, allowing it to play a regulatory function that suppresses or augments serotonin neurotransmission depending on downstream neurotransmission (Albert et al., 2011). Given the existence of numerous small molecule therapies capable of targeting individual 5HT receptors classes, better characterization of serotoninergic therapeutic targets in iRBD has the potential to quickly translate into 5HT-focused clinical trials.

Limitations of our study include the small sample size, demographic factor differences between iRBD participants and older controls, and the absence of detailed uniform clinical assessments. It is also important to note that our data set is cross-sectional only and cannot provide insight into longitudinal risk for differential conversion to either PD, DLB or MSA. Studies involving larger cohorts would also afford the statistical power to control for confounders as covariates using regression models. We would also note that analyses presented in Table 1 did not adjust or multiple comparisons, leaving open the possibility of type 1 error. Given these limitations, our findings should be interpreted as exploratory and hypothesis generating in nature. Despite these limitations, DASB PET is a rigorous and well-characterized imaging approach that has been used in other common psychiatric and neurodegenerative conditions.

Based on the role of the serotonin system in sleep and as a risk factor for RSWA, the addition of serotonin-specific biomarkers to future iRBD natural history studies already has strong rationale. DASB PET findings from this study provide further support for this and raise the possibility that therapeutic targets within the serotoninergic nervous system may deserve careful scrutiny for neuroprotective synucleinopathy trials moving forward.

## Data availability statement

Deidentified data supporting the conclusions of this article will be made available by the authors, without undue reservation.

## Ethics statement

The studies involving humans were approved by University of Michigan IRBMED A2 Board. The studies were conducted in accordance with the local legislation and institutional requirements. iRBD participants provided their written informed consent to participate in this study.

## Author contributions

MG: Funding acquisition, Investigation, Writing – review & editing. PV: Writing – original draft, Writing – review & editing. NB: Writing – review & editing, Investigation, Methodology, Resources. RK: Investigation, Methodology, Resources, Writing – review & editing. VK: Conceptualization, Data curation, Formal analysis, Funding acquisition, Investigation, Methodology, Writing – original draft.
